# Yogi Detox Tea: A Potential Cause of Acute Liver Failure

**DOI:** 10.1155/2017/3540756

**Published:** 2017-10-24

**Authors:** Keerthana Kesavarapu, Mitchell Kang, Jaewook James Shin, Kenneth Rothstein

**Affiliations:** ^1^Department of Internal Medicine, Drexel University Hospital, Philadelphia, PA, USA; ^2^Drexel School of Medicine, Philadelphia, PA, USA; ^3^Department of Gastroenterology, Drexel University Hospital, Philadelphia, PA, USA; ^4^Department of Hepatology, Drexel University Hospital, Philadelphia, PA, USA

## Abstract

We present a case of acute fulminant liver failure from a liver detoxification tea. We present a 60-year-old female with weakness, lethargy, scleral icterus, jaundice, and worsening mental status. She drank herbal tea three times a day for 14 days prior to symptom development. Liver tests were elevated. Remaining laboratory tests and imaging were negative for other etiologies. An ultrasound-guided liver biopsy showed submassive necrosis. A literature search on the ingredients shows six ingredients as having hepatotoxic effects and remaining ingredients as having very sparse hepatoprotective data. Healthcare professionals should discuss herbal medication and tea use and report adverse effects.

## 1. Introduction

The American Association for the Study of Liver Diseases (AASLD) defines acute fulminant liver failure as an acute deterioration of function resulting in altered mentation and coagulopathy without any known preexisting disease [1]. In 308 patients from 17 tertiary care centers participating in US Acute Liver Failure Study Group, drug reactions including acetaminophen overdose were the presumptive cause in 52% of cases [2]. Prevalent medication offenders include acetaminophen, mushrooms, and antiepileptics (phenytoin and valproic acid); however herbal supplement induced hepatotoxicity is gaining prevalence. According to the Drug-Induced Liver Injury Network (DILIN), causes of acute liver failure (ALF) from herbal supplements have increased from 7% to 20% over their study period from 2007 to 2013 [3]. This number is probably a gross underestimation as patients do not report use to their physicians and physicians do not report side effects to the Food and Drug Association (FDA) and might not be identified by DILIN. Here we report a case of ALF after the use of an herbal detoxification tea marketed to be hepatoprotective.

## 2. Case Report

A 60-year-old female with past medical history of hypertension presented with new-onset generalized weakness and lethargy worsening over the past two weeks. She denied any fevers, chills, changes in stool, changes in mental status, sick contacts, or recent travel. Her past medical history was significantly only for obesity. Her social history included no tobacco use, frequent alcohol consumption (3 glasses of wine every night), no IV drug use, no acetaminophen use, and no high risk sexual activity. Her alcohol use has been stable since she started drinking ten years ago. Previous laboratory testing showed normal liver function. She was fully vaccinated with no history of hepatitis. Her only home medication was hydrochlorothiazide, which she had been taking for years. She had no preexisting liver disease with normal liver function tests prior to this admission. She reported drinking Yogi Detox herbal tea three times a day for 14 days prior to symptom development. As per the patient, she was consuming this tea as a cleanse.

Physical examination demonstrated a normotensive and afebrile patient in mild distress. the patient was jaundiced with scleral icterus. Examination demonstrated a soft and nondistended abdomen with moderate right upper quadrant tenderness. Mental status was intact on admission; however, on the ninth day of the admission she became lethargic and developed asterixis. Initial laboratory tests are depicted in Table 1. Radiological examinations performed consisted of an abdominal ultrasound with Doppler's and triple-phase computerized tomography (CT) with contrast of the abdomen, which were normal. Extensive laboratory testing was ordered to determine the etiology of her liver failure. Serological markers including those for autoimmune hepatitis (Anti-KLM antibodies, ANA, and AMA), viral hepatitis (A, B, C, and D), Wilson's disease (ceruloplasmin), and alpha-1-antitrypsin deficiency were analyzed and found to be negative. In addition, CMV, EBV, VZV, and HSV were negative. HEV testing was not performed as there is no approved test in the United States [4].

There was concern that the cause of her liver injury might have been due to her Yogi detox herbal tea consumption. Using the 2016 Roussel Uclaf Causality Assessment Method (RUCAM) score as highlighted in Table 2, the relationship between this herbal tea and liver injury was determined [26].

**Figure 1:**
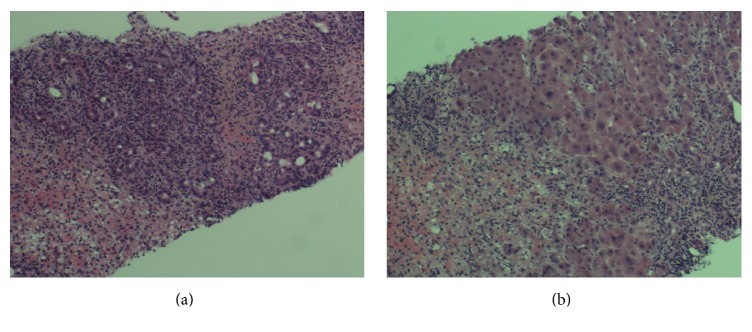
US-guided liver biopsy showing submassive necrosis.

The *R* ratio, which is the initial step in the RUCAM assessment, was 8.16 indicating a hepatocellular pattern of injury. In our patient, her RUCAM score was 7 (2 points for time from drug intake < 15 days, 2 points for ≥50% reduction of ALT after herb cessation, 1 point for risk factors of alcohol use, 2 points for other causes being ruled out, and 1 point for previous reaction to herb but unlabeled), which indicates that the detox tea is the probable offending agent of her hepatic injury. Given the worsening clinical picture, an ultrasound-guided liver biopsy was performed. The liver histology was notable for submassive necrosis with portal, periportal, and panlobular inflammation with lymphocytes, numerous neutrophils, plasma cells, and few eosinophils (Figures 1(a) and 1(b)). Hepatocyte ballooning, Mallory hyaline, and single cell apoptosis were also noted. Her lethargy progressed to somnolence requiring intubation. She was placed on the transplant list on day 15 and passed away on day 17.

## 3. Discussion

The prevalence of herbal supplementation intake has been increasing; however, their use is unregulated by the Food and Drug Administration and unsupervised by medical professionals [27]. The patient discussed in this case consumed Yogi Detoxification tea, an American produced tea that is an amalgamation of eighteen herbs marketed to be hepatoprotective. The Ingredients of this tea are listed in Table 3. Literature review of these ingredients in PubMed showed that all had articles endorsing hepatoprotection. The basis behind protection is hypothesized and demonstrated to be through their antioxidant and anti-inflammatory properties. However, literature search of these ingredients showed that less than 10% were done with human or liver-injury cell models. Animal studies may not be able to mimic the complex process of herb-induced inflammatory response of human physiology and progress. Therefore, the safety and efficacy may not be translatable to human trials. Not only is more basic research needed to understand the pathophysiology of hepatotoxicity, but more human trials are necessary to elucidate potential effects.

Six ingredients had one or more published studies associating it with hepatotoxicity. Hepatic toxicity caused by Gardenia has been demonstrated to cause oxidative stress-induced hepatocyte necrosis and apoptosis in murine models. Seven different articles attributed Gardenia's component geniposide to its hepatotoxic effect [19–25]. Yang described liver biopsy findings in rats exposed to geniposides that showed swollen and necrotic cells and an inflammatory infiltrate similar to the biopsy of our patient [25]. Similarly, skullcap root, another ingredient, was shown in a two-year retrospective study of 1169 hospitalized patients to be a common cause of hepatotoxicity [12]. Four case reports featuring seven patients show complications can range from mild hepatitis to acute hepatic failure requiring transplantation [13–16]. Cinnamon bark, black pepper, juniper berry, and rhubarb root, other ingredients of this tea, have all been associated with liver dysfunction [5–7].

While evidence suggests that the patient's acute change is associated with tea consumption, the exact toxicity-inducing agent of this tea is unclear. Preexisting alcohol consumption in combination with the herbal supplementation may have hastened the progression of hepatotoxicity. In addition to the 19 ingredients, herbal products are contaminated with other toxins or added adulterants that are not advertised on the packaging. In addition, ingestion of multiple substances may increase the risk of hepatotoxicity. This “cluster” effect of herbal supplements has not been elucidated in the literature; however, it has been known that risk factors such as alcohol ingestion are susceptible to drug toxicity from alterations in drug metabolism. Regardless, herbal tea consumption must be monitored by healthcare professionals, and any side effects should be reported to specific organizations in order to take regulatory measures and control usage of such products.

## Figures and Tables

**Table 1:** Hepatic function panel on day of admission, 1 week and 2 weeks later.

Laboratory testing	On admission	1 week	2 weeks
Aspartate aminotransferase (5–35 U/L)	450	1864	51
Alanine aminotransferase (4–35 U/L)	583	2162	82
Alkaline phosphatase (30–99 U/L)	202	143	72
Total bilirubin (0.2–1.2 mg/dl)	27	43	30
Direct bilirubin (0.01–0.19 mg/dl)	20	33	21
INR (0.9–1.2)	4	2.7	7.8

**Table 2:** Roussel Uclaf Causality Assessment Method, an assessment of the causality of drug-induced liver injury.

Items for mixed liver injury	Score in our patient
Time to onset from cessation of drug/herb: <15 days	+2
Course of ALT after cessation of drug/herb: decrease ≥ 50% within 180 days	+2
Risk factors: alcohol use (>2 drinks/d for women) and Age ≥ 55	+1
Concomitant drug/herbs: none	0
Alternative causes: all causes ruled out	+2
Previous hepatotoxicity of the drug/herb: reaction published but unlabeled	+1
Response to unintentional reexposure: none	0
Total score	8

**Table 3:** 18-ingredient list contained in the Yogi Detox Tea and the articles published in PubMed highlighting their hepatotoxicity.

Ingredient	Number of hepatotoxic articles/case reports
Sarsaparilla root	0
Cinnamon bark	1 [5]
Ginger root	0
Licorice root	0
Dandelion root	0
Cardamom seed	0
Clove bud	0
Black pepper	1 [6]
Juniper berry	1 [7]
Long pepper berry	0
Phellodendron bark	0
Rhubarb root	4 [8–11]
Skullcap root	7 [12–18]
Coptis root	0
Forsythia fruit	0
Gardenia fruit	7 [19–25]
Honeysuckle	0
Winter melon	0
